# Causal effects of modifiable risk factors on kidney stones: a bidirectional mendelian randomization study

**DOI:** 10.1186/s12920-023-01520-z

**Published:** 2023-04-20

**Authors:** Wen Liu, Miaomiao Wang, Jianyong Liu, Qiuxia Yan, Ming Liu

**Affiliations:** 1 Department of Urology, Beijing Hospital, National Center of Gerontology, Institute of Geriatric Medicine, Chinese Academy of Medical Sciences, Beijing, China; 2 Graduate School of Peking Union Medical College, Chinese Academy of Medical Sciences, Beijing, China; 3 Fifth School of Clinical Medicine, Peking University, Beijing, China

**Keywords:** Mendelian randomization, Kidney stones, Risk factors, Causality

## Abstract

**Background:**

Increasing epidemiological studies demonstrated that modifiable risk factors affected the risk of kidney stones. We aimed to systemically assess these causal associations using a bidirectional Mendelian randomization study.

**Methods:**

We obtained instrumental variables related to each exposure at the genome-wide significant threshold (P < 5 × 10^–8^). Summary level data for outcomes from the FinnGen consortium and UK Biobank were utilized in the discovery and replication stage. The Inverse-variance weighted (IVW) method was used as the primary analysis, with additional sensitivity analyses and fix-effect meta-analysis to verify the robustness of IVW results.

**Results:**

Among 46 risk factors, five were significantly associated with nephrolithiasis risk in the FinnGen consortium, UK Biobank, and meta-analyses collectively. The odds ratios (ORs) (95% confidence intervals [95%CIs]) of kidney stones were 1.21 (1.13, 1.29) per standard deviation (SD) increase in serum calcium, 1.55 (1.01, 2.36) per SD increase in serum 25(OH)D, 1.14 (1.00, 1.29) per SD increase in total triglycerides, 2.38 (1.34, 4.22) per SD increase in fasting insulin, and 0.28 (0.23, 0.35) per unit increase in log OR of urine pH. In addition, genetically predicted serum phosphorus, urinary sodium, tea consumption, and income affected the risk of kidney stones (false discovery rate [FDR] *P* < 0.05) based on the outcome data from the FinnGen consortium, and the significant associations of education and waist-to-hip ratio with nephrolithiasis risks were found after FDR correction (FDR *P* < 0.05) based on the outcome data from UK Biobank.

**Conclusions:**

Our findings comprehensively provide modifiable risk factors for the prevention of nephrolithiasis. Genome-wide association studies with larger sample sizes are needed to verify these causal associations in the future further.

**Supplementary Information:**

The online version contains supplementary material available at 10.1186/s12920-023-01520-z.

## Introduction

Kidney stone disease is a common cause of morbidity, with nearly 8.8% incidence in the United States, and poses a high economic burden globally [1]. Surgical treatments only remove the existing stone and do little to decrease the frequent recurrence of kidney stones. Therefore, identifying modifiable risk factors to reduce stone formation is necessary and has sparked increasing interest in recent years.

In most individuals, the underlying etiology of kidney stones is considered multifactorial, including environmental and genetic factors [2]. Many epidemiological studies aimed to discover potentially modifiable risk factors that could be modulated to reduce the incidence of kidney stones, involving obesity [3], cardiometabolic related factors (hypertension, dyslipidemia, type 2 diabetes [T2D], and glycemic traits) [4, 5], diet [6], lifestyle [7], and blood and urine minerals [1]. Nevertheless, most of the studies were conventional observational studies, which were insusceptible to demonstrating causality due to inherent methodological biases and reverse causation [8]. In addition, due to the high time and expense of randomized controlled trials, these trials are scarce and sometimes limited when implementing interventions.

Mendelian randomization (MR) is one way to assess causality, which utilizes genetic variants (single-nucleotide polymorphisms [SNPs]) robustly connected with exposures as instruments to explore the causal associations of exposures with outcomes. By taking advantage of the random assortment of SNPs at conception, MR studies are less vulnerable to confounding and reverse causation bias than conventional observational studies [9]. Several MR analyses have explored the associations between single modifiable risk factors and kidney stones, such as obesity [10], T2D [10], cardiovascular events [11], coffee and caffeine consumption [12], education [13], serum urate [14], 25(OH)D [15], and calcium [16]. Here we aimed to extend our analysis to comprehensively estimate the causal effects of 47 potentially modifiable risk factors on the risk of kidney stones by using a bidirectional MR approach.

## Methods

### Study design overview

**Fig. 1:**
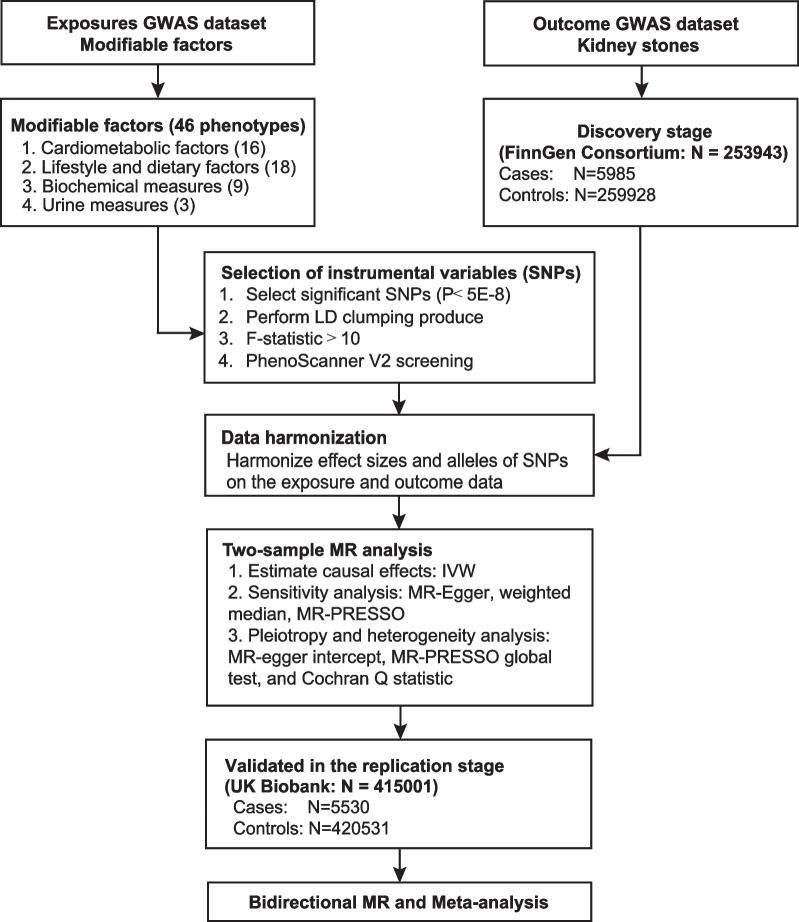
Diagrammatic description of the process of MR analysis in this study. GWAS, genome-wide association studies; SNP, single nucleotide polymorphism; LD, linkage disequilibrium; MR, Mendelian randomization; IVW, inverse variance weighting; MR-PRESSO, Mendelian randomization pleiotropy residual sun and outlier

We firstly conducted a systematic review through the PubMed database to identify all potential factors for kidney stones and some modifiable factors that might be associated with kidney stones (up to 10 May 2022). The search terms and potential risk factors are provided in Additional file 1: Table S1. We included 46 factors on the basis: (1) a potentially modifiable risk factor, (2) publicly available genome-wide association studies (GWASs) or summary level data, (3) the number of instrument variables (IVs) ≥ 3. To assess the causal relationships of modifiable risk factors with kidney stones, we performed two-sample MR (TSMR) using summary statistics from the FinnGen consortium (the discovery stage) and UK Biobank (the replication stage) and conducted bidirectional TSMR for the significant risk factors from TSMR with available summary statistics. The procedure of MR is illustrated in Fig. 1.

### Genetic instrumental variables for modifiable risk factors

The included risk factors can be categorized into four groups: cardiometabolic factors, lifestyle and dietary factors, biochemical measures, and urine measures. We searched PubMed and consortia for GWASs of the modifiable risk factors and identified genetic variants from non-UK Biobank or UK Biobank GWAS in individuals of European ancestry. Exposure GWASs from UK Biobank were only used in the MR analysis of the discovery stage. Details of exposure GWASs are shown in Table 1 and Additional file 1: Table S2.

**Table 1:** Description of modifiable risk factors in the discovery stage

Traits	Consortium or Study	Number of SNPs	Sample size	Unit	Variance explained (%)	PMID
Cardiometabolic factors						
BMI	GIANT	60	322,154	SD	1.14	25,673,413
WHR adjusted BMI	GIANT	36	210,088	SD	0.94	25,673,412
Height	GIANT	357	253,288	SD	10.95	25,282,103
Body fat percentage	–	5	89,297	SD	0.23	26,833,246
LDL cholesterol	GLGC	286	842,635	SD	5.08	34,887,591
Total triglycerides	GLGC	337	864,202	SD	4.72	34,887,591
Total cholesterol	GLGC	338	930,666	SD	5.69	34,887,591
HDL cholesterol	GLGC	381	888,220	SD	6.18	34,887,591
Fasting glucose	MAGIC	64	200,622	SD	3.72	34,059,833
Fasting insulin	MAGIC	35	151,013	SD	1.19	34,059,833
Glycated hemoglobin (HbA1c)	MAGIC	70	146,864	SD	5.40	34,059,833
2 h glucose	MAGIC	9	63,578	SD	1.11	34,059,833
Type 2 diabetes mellitus	DIAGRAM	86	455,313	Log-odds	1.27	30,297,969
Systolic blood pressure	ICBP	10	299,024	10 mmHg	0.13	30,224,653
Diastolic blood pressure	ICBP	14	299,024	10 mmHg	0.19	30,224,653
Metabolic syndrome	UK Biobank	60	291,107	Log-odds	1.97	31,589,552
Lifestyle and dietary factors						
Smoking cessation	GSCAN	4	143,851	Log-odds	0.12	30,643,251
Smoking initiation (ever regular vs. never regular)	GSCAN	10	249,171	Log-odds	0.15	30,643,251
Smoking cigarettes per day	GSCAN	9	143,210	SD	0.65	30,643,251
Alcohol drinks per week	GSCAN	5	226,223	Drinks/week	0.08	30,643,251
Years of education	SSGAC	47	293,723	SD	0.64	27,225,129
Caffeine consumption from Tea, mg/day	UK Biobank	22	395,866	SD	0.25	33,287,642
Caffeine consumption from coffee or tea, mg/day	UK Biobank	41	362,316	SD	0.84	33,287,642
Glucocorticoids (medication use)	UK Biobank	19	205,700	SD	0.63	31,015,401
Accelerometer-based PA (acceleration average)	UK Biobank	8	91,084	SD	0.30	29,899,525
Moderate-to-vigorous PA	UK Biobank	17	377,234	SD	0.15	29,899,525
Vigorous PA	UK Biobank	7	261,055	SD	0.11	29,899,525
Income before tax	UK Biobank	25	286,301	SD	0.35	31,844,048
Intelligence	UK Biobank	157	269,867	SD	2.50	29,942,086
Leisure sedentary behavior (computer use)	UK Biobank	45	408,815	SD	0.43	32,317,632
Driving	UK Biobank	4	408,815	SD	0.04	32,317,632
Leisure sedentary behavior (television watching)	UK Biobank	125	408,815	SD	1.31	32,317,632
Meat-related diet	UK Biobank	14	335,576	SD	0.14	32,066,663
Fish and plant-related diet	UK Biobank	22	335,576	SD	0.32	32,066,663
Biochemical measures						
Serum vitamin B12	Icelandic + Danish	9	45,576	SD	5.00	23,754,956
Serum Vitamin C	–	7	52,018	SD	1.29	33,203,707
Serum 25-hydroxyvitamin D	SUNLIGHT	6	79,366	SD	2.23	29,343,764
Serum magnesium	CHARGE	5	23,829	SD	1.55	20,700,443
Serum calcium	–	5	61,011	SD	0.88	24,068,962
Serum phosphorus	CHARGE	5	21,734	SD	1.30	20,558,539
Serum CRP	CHARGE	17	204,402	SD	0.87	30,388,399
Serum uric acid	GUGC	15	110,347	SD	1.46	23,263,486
Serum adiponectin	ADIPOGen	13	29,347	SD	8.78	22,479,202
Urine measures						
Urine pH (pH < = 5.0 vs pH > 5.0)	Iceland	3	148,199	Log-odds	0.10	30,476,138
Urinary sodium	UK Biobank	48	446,237	SD	0.46	31,409,800
Urinary potassium	UK Biobank	20	446,230	SD	0.17	31,409,800

*SNPs* single nucleotide polymorphisms, *PMID* the publication ID in PubMed, *SD* standard deviation, *BMI* body mass index, *WHR* waist-to-hip ratio, *2-h glucose* the 2-h glucose level of the oral glucose tolerance test, *PA* physical activity, *LDL* low-density lipoprotein, *HDL* high-density lipoprotein, *CRP* C-reactive protein, *GIANT* Genetic Investigation of Anthropometric Traits, *GLGC* Global Lipids Genetics Consortium, *MAGIC* the Meta-Analyses of Glucose and Insulin-related traits Consortium, *DIAGRAM* DIAbetes Genetics Replication And Meta-analysis, *ICBP* International Consortium for Blood Pressure, *GSCAN* the GWAS and Sequencing Consortium of Alcohol and Nicotine use, *SSGAC* the Social Science Genetic Association Consortiumv*SUNLIGHT* Study of Underlying Genetic Determinants of Vitamin D and Highly Related Traits, *CHARGE* the Cohorts for Heart and Aging Research in Genomic Epidemiology, *GUGC* Global Urate Genetics Consortium

We implemented a series of quality control steps to select IVs. First, we identified SNPs related to each risk factor at the genome-wide significant threshold (*P* < 5 × 10^–8^) as IVs. For each SNP, only those with minor allele frequency (MAF) greater than 0.01 were available for subsequent analyses. Second, correlated SNPs were clumped at a threshold of linkage disequilibrium (LD) r^2^ > 0.001 and a distance of 10,000 kb, with SNPs with the lowest *P*-value retained. Third, we calculated the F-statistic of each SNP to assess its strength as previously described [17], with F-statistic < 10 indicating weak instrument bias. Fouth, we utilized the PhenoScanner V2 to check and remove genetic variants that exhibit significant associations with different phenotypes, thus preventing the possible horizontal pleiotropy [18, 19]. Details of IVs were presented in Additional file 1: Table S4–S5.

### GWAS summary statistics for kidney stones

Summary statistics for nephrolithiasis from the sixth release of the FinnGen consortium [20] and UK Biobank [21] were used in the discovery and replication stages, respectively, considering the proportion of cases in the FinnGen consortium is relatively higher. In the FinnGen consortium, patients with kidney stones were defined by N20 in the International Classification of Diseases, 10th Revision (ICD-10) and 592 in ICD-8 and ICD-9. This GWAS was performed on 5,985 cases and 253,943 controls with the adjustment for sex, age, first ten principal components, genotyping batch, and genetic relatedness. In UK Biobank, patients were defined by N20 in ICD-10. A total of 5,530 cases and 415,001 controls were included after excluding the individuals of non-European ancestry, with adjusting age, sex, age squared, the interaction between sex and age, the interaction between sex and age squared, and the first ten principal components. Details of the outcome GWASs were presented in Additional file 1: Table S3.

### Statistical analysis

Three assumptions are required for the MR approach: (1) genetic variants must robustly connect with exposure (modifiable risk factors); (2) genetic variants should be independent of confounders; (3) genetic variants can only affect outcomes (the risk of kidney stones) through exposure.

The random-effects inverse-variance weighted (IVW) method was applied as the primary MR analysis [22]. The IVW method will return an unbiased estimate without horizontal pleiotropy or when horizontal pleiotropy is balanced. Then, sensitivity analyses were performed using weighted-median estimation [23], MR-Egger regression analysis [24], and the MR pleiotropy residual sum and outlier (MR-PRESSO) test [25]. The weighted-median method provides robust estimates if less than 50% of the weight is pleiotropic. MR-Egger regression analysis provides unbiased causal estimates even though the genetic variants violate the third assumptions. These methods hold different assumptions at the cost of reduced statistical power. In addition, MR results may be biased by horizontal pleiotropy. The MR-Egger method can produce an intercept term to detect directional pleiotropy. When the intercept *P* value is > 0.05, the directional pleiotropy is not present [24]. MR-PRESSO test was applied to detect directional pleiotropic outliers and to eliminate the effects of pleiotropy by removing outliers [25]. Cochran's Q test was performed to assess the heterogeneity in the IVW method.

Effect estimates were reported as odds ratios (OR) with 95% confidence intervals (CI) per unit increase in each risk factor (Table 1 and Additional file 1: Table S2). In addition, we performed fixed-effect meta-analyses to combine the IVW results derived from the FinnGen consortium and UK Biobank. A false discovery rate (FDR) correction was used in the IVW method to adjust for multiple testing. An FDR corrected *P*-value < 0.05 was considered statistically significant with solid causal evidence, and the uncorrected IVW *P*-value < 0.05 was regarded as evidence of a suggestive association. However, we interpreted the evidence based on the statistical significance, the consistency of the results (FinnGen consortium and UK Biobank), and the effect estimates of meta-analyses. A modifiable factor is considered significantly related to nephrolithiasis risk if it shows a statistical significance in either the FinnGen consortium or UK Biobank with an FDR corrected *P*-value < 0.05, or in the FinnGen consortium, UK Biobank, and meta-analyses collectively with an IVW *P*-value < 0.05. The statistical power for MR was calculated based on the website (mRnd) [26].

All statistical analyses were performed with “TwosampleMR,” “MR-PRESSO,” and “Meta” packages [27] in R 4.1.1 (R Foundation for Statistical Computing, Vienna, Austria). All data were publicly downloadable from GWASs or summary statistics without individual-level data, and ethical approval was obtained in the original studies.

## Results

### Discovery results based on the FinnGen consortium

In the discovery stage, 11 of 46 modifiable risk factors were causally associated with the risk of kidney stones. Genetically predicted lower levels of caffeine consumption from tea, income, serum phosphorus, and urine pH, whereas higher levels of serum calcium, fasting insulin, and urinary sodium could increase the risk of kidney stones after FDR correction (FDR *P* < 0.05). The ORs (95%CIs) of kidney stones decreased per standard deviation (SD) increase in caffeine consumption from tea (0.30 [0.14, 0.67]), income (0.24 [0.10, 0.59]), and serum phosphorus (0.47 [0.28, 0.78]), and per unit increase in log OR of urine pH (0.28 [0.23, 0.35]). For per SD increase in serum calcium, fasting insulin, and urinary sodium, the ORs (95%CIs) of kidney stones were 1.21 (1.13, 1.29), 2.38 (1.34, 4.22), and 3.46 (1.59, 7.53), respectively. In addition, two risk factors suggestively elevated the risk of kidney stones (OR [95% CI] per SD increase in total triglycerides 1.14 [1.00, 1.29] and 25(OH)D 1.55 [1.01, 2.36]) (Table 2 and Additional file 1: Table S6). However, because of limited evidence (FDR *P* > 0.05 and IVW *P* < 0.05) and not validated in the replication stage, we deemed that serum vitamin B12 and magnesium were not associated with nephrolithiasis risk. Finally, we conducted reverse MR analyses and found that kidney stones significantly increased the risk of systolic and diastolic blood pressure (Additional file 1: Table S9).

**Table 2:** Mendelian randomization results for the associations between genetically predicted risk factors and kidney stones in two stages

Modifiable factors^a^	Number of SNPs^b^	Discovery stage^c^			Replication stage^d^		
		IVW OR (95% CI)	*P*-value	FDR^e^	IVW OR (95% CI)	*P*-value	FDR^e^
Cardiometabolic factors							
BMI	60	0.99 (0.77, 1.28)	0.951	0.972	1.16 (0.93, 1.45)	0.184	0.502
WHR adjusted BMI	36	1.08 (0.84, 1.39)	0.529	0.737	**1.5 (1.16, 1.94)**	**0.002**	**0.020**
Height	357	0.94 (0.86, 1.02)	0.123	0.319	**0.91 (0.83, 0.99)**	**0.031**	0.116
Body fat percentage	5	0.72 (0.39, 1.33)	0.292	0.537	1.02 (0.28, 3.66)	0.979	0.979
LDL cholesterol	286	1.05 (0.93, 1.18)	0.430	0.659	0.95 (0.85, 1.06)	0.369	0.692
Total triglycerides	337	**1.14 (1, 1.29)**	**0.048**	0.201	**1.12 (1.00, 1.26)**	**0.046**	0.138
Total cholesterol	338	1.11 (0.98, 1.24)	0.091	0.279	1.02 (0.91, 1.15)	0.695	0.825
HDL cholesterol	381	1.02 (0.91, 1.15)	0.676	0.808	1.03 (0.92, 1.15)	0.627	0.825
Fasting glucose	64	1.11 (0.86, 1.42)	0.427	0.659	1.21 (0.9, 1.64)	0.214	0.519
Fasting insulin	35	**2.38 (1.34, 4.22)**	**0.003**	**0.020**	**3.01 (1.8, 5.03)**	**2.54E−05**	**0.001**
Glycated hemoglobin (HbA1c)	70	0.94 (0.65, 1.36)	0.731	0.820	1.21 (0.83, 1.77)	0.312	0.669
2 h glucose	9	0.93 (0.68, 1.27)	0.645	0.808	0.98 (0.72, 1.34)	0.889	0.953
Type 2 diabetes mellitus	86	1.04 (0.97, 1.1)	0.287	0.537	**1.08 (1, 1.16)**	**0.046**	0.138
SBP	10	1 (0.95, 1.04)	0.903	0.948	1 (0.94, 1.07)	0.950	0.979
DBP	14	0.95 (0.9, 1.01)	0.079	0.279	0.98 (0.93, 1.04)	0.612	0.825
Metabolic syndrome	60	0.94 (0.85, 1.04)	0.217	0.454	–	–	–
Lifestyle and dietary factors							
Smoking cessation	4	1.03 (0.66, 1.62)	0.897	0.948	**1.18 (1.04, 1.33)**	**0.008**	**0.040**
Smoking initiation (ever regular vs never regular)	10	0.98 (0.74, 1.31)	0.907	0.948	0.9 (0.54, 1.51)	0.700	0.825
Smoking cigarettes per day	9	1.29 (0.93, 1.79)	0.125	0.319	0.88 (0.62, 1.24)	0.453	0.799
Alcohol drinks per week	5	2.33 (0.83, 6.48)	0.106	0.305	0.63 (0.25, 1.62)	0.336	0.672
Years of education	47	0.79 (0.56, 1.1)	0.167	0.366	**0.7 (0.53, 0.92)**	**0.011**	**0.047**
Caffeine consumption from Tea	22	**0.3 (0.14, 0.67)**	**0.003**	**0.020**	–	–	–
Caffeine consumption from coffee or tea	41	0.82 (0.59, 1.14)	0.232	0.464	–	–	–
Glucocorticoids (medication use)	19	0.94 (0.87, 1.01)	0.086	0.279	–	–	–
Accelerometer-based PA (acceleration average)	8	1.03 (0.94, 1.13)	0.564	0.741	–	–	–
Moderate-to-vigorous PA	17	1 (0.38, 2.64)	0.992	0.992	–	–	–
Vigorous PA	7	0.49 (0.06, 3.77)	0.494	0.733	–	–	–
Income before tax	25	**0.24 (0.1, 0.59)**	**0.002**	**0.020**	–	–	–
Intelligence	157	0.86 (0.71, 1.05)	0.140	0.322	–	–	–
Leisure sedentary behavior (computer use)	45	0.88 (0.51, 1.53)	0.660	0.808	–	–	–
Driving	4	1.25 (0.98, 1.59)	0.067	0.257	–	–	–
Leisure sedentary behavior (television watching)	125	1.12 (0.88, 1.43)	0.363	0.596	–	–	–
Meat-related diet	14	0.78 (0.36, 1.69)	0.528	0.737	–	–	–
Fish and plant-related diet	22	1.22 (0.63, 2.34)	0.553	0.741	–	–	–
Biochemical measures							
Serum vitamin B12	9	**1.16 (1.01, 1.34)**	**0.039**	0.198	0.99 (0.86, 1.13)	0.843	0.937
Serum Vitamin C	7	1.12 (0.97, 1.3)	0.134	0.322	1.4 (0.81, 2.41)	0.225	0.519
Serum 25-hydroxyvitamin D	6	**1.55 (1.01, 2.36)**	**0.043**	0.198	**1.56 (1.13, 2.16)**	**0.007**	**0.040**
Serum magnesium	5	**1.81E2 (2.57, 1.28E4)**	**0.017**	0.098	4.84 (0.01, 2112.4)	0.611	0.825
Serum calcium	5	**1.21 (1.13, 1.29)**	**1.52E−08**	**3.50E−07**	**1.55 (1.21, 1.99)**	**0.001**	**0.015**
Serum phosphorus	5	**0.47 (0.28, 0.78)**	**0.003**	**0.020**	0.88 (0.43, 1.77)	0.715	0.825
Serum CRP	17	0.88 (0.68, 1.15)	0.351	0.596	1.11 (0.81, 1.52)	0.526	0.825
Serum uric acid	15	0.96 (0.78, 1.19)	0.730	0.820	0.97 (0.82, 1.15)	0.706	0.825
Serum adiponectin	13	1.05 (0.82, 1.36)	0.685	0.808	1.07 (0.81, 1.41)	0.653	0.825
Urine measures							
Urine pH (pH pH > 5.0 VS ≤ 5.0)	3	**0.28 (0.23, 0.35)**	**2.72E−29**	**1.25E−27**	**0.26 (0.1, 0.68)**	**0.006**	**0.040**
Urinary sodium	48	**3.46 (1.59, 7.53)**	**0.002**	**0.020**	–	–	–
Urinary potassium	20	2.15 (0.45, 10.39)	0.341	0.596	–	–	–

A two-sided p-value < 0.05 was considered statistically significant and shown in bold

*SNP* single-nucleotide polymorphism, *IVW* inverse-variance weighted, *FDR* false discovery rates, *OR* odds ratio, *CI* confidence interval, *SBP* Systolic blood pressure, *DBP* Diastolic blood pressure, *WHR* waist-hip ratio, *BMI* body mass index, *HDL* high-density lipoprotein, *LDL* low-density lipoprotein, *HDL* high-density lipoprotein, *PA* physical activity, *CRP* C-reactive protein

^a^If the genetic instruments for exposures were obtained from UK Biobank, these exposures were not validated in the replication stage

^b^SNPs represent the number of SNPs used within the instrument for each exposure after clumping, harmonization, and data extraction from the included GWAS

^c^The summary level data were extracted from the FinnGen consortium

^d^The summary level data were extracted from UK Biobank

^e^An FDR corrected *P*-value < 0.05 was considered statistically significant, indicating strong evidence of causality

Sensitivity analyses confirmed the robustness of the results. The MR-Egger method showed no evidence of horizontal pleiotropy in the above 11 modifiable factors (the intercept P-value > 0.05). However, there were heterogeneity and outliers in total triglycerides and income. Thus, we removed the outlying SNPs and showed consistent results in the corrected MR-PRESSO method (Additional file 1: Table S6). Furthermore, no significant causal relationship was found between other modifiable risk factors and kidney stones.

### Validation results based on UK Biobank

In the replication stage, we identified 10 modifiable risk factors causally related to the risk of kidney stones from 30 risk factors. Consistent with the IVW results of the replication stage, MR results successfully validated the significant association and similar direction of total triglycerides, fasting insulin, serum 25(OH)D, calcium, and urine pH with the risk of kidney stones (Table 2). In addition, genetically predicted higher levels of waist-to-hip ratio adjusted body mass index (WHRadjBMI), fasting insulin, serum 25(OH)D, and serum calcium, whereas lower levels of years of education, and urine pH could elevate nephrolithiasis risk after FDR correction (FDR *P* < 0.05). Reverse MR analyses did not discover the significant impact of kidney stones on the above risk factors with summary statistics (Additional file 1: Table S9). Heterogeneity and outliers were still found in total triglycerides. After removing outliers, the corrected MR-PRESSO results remained significant (Additional file 1: Table S7).

Among these 10 risk factors, there was limited evidence supporting the causal associations of height, and T2D with the risk of kidney stones, which were not only FDR *P* > 0.05 and IVW *P* < 0.05 but also not validated in the FinnGen consortium. Due to low statistical power (0.07), we deemed that the relationship between smoking cessation and kidney stones needs further investigation.

### Meta-analyses based on FinnGen and UK Biobank

**Fig. 2:**
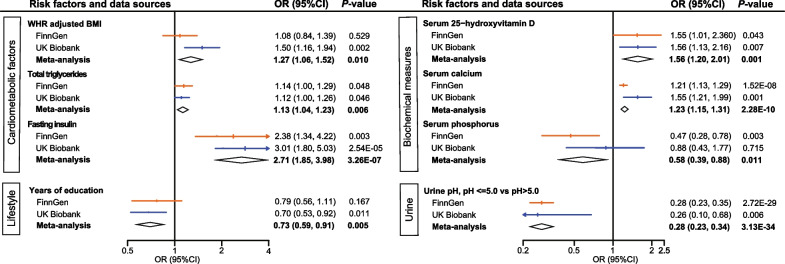
Meta-analyses of MR results from both the FinnGen consortium and UK Biobank. MR, Mendelian randomization; OR, odds ratio; CI, confidence interval; BMI, body mass index

We performed meta-analyses of IVW results from two sources to further confirm the significant impact of modifiable risk factors on nephrolithiasis risk, including WHRadjBMI (OR = 1.27, *P* = 0.010), total triglycerides (OR = 1.13, *P* = 0.006), fasting insulin (OR = 2.71, *P* = 3.26 × 10^–07^), serum 25(OH)D (OR = 1.56, *P* = 0.001), serum calcium (OR = 1.23, *P* = 2.28 × 10^–10^), serum phosphorus (OR = 0.58, *P* = 0.011), years of education (OR = 0.73, *P* = 0.005), and urine pH (OR = 0.28, *P* = 3.13 × 10^–34^)(Fig. 2).

## Discussion

**Fig. 3:**
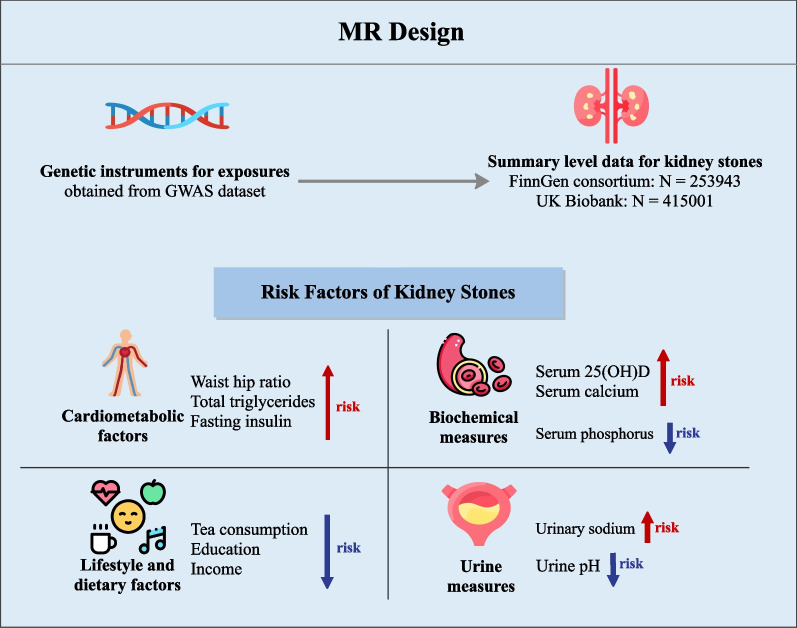
Genetically predicted risk factors for kidney stones. MR, Mendelian randomization; GWAS, genome-wide association studies

Given that the underlying etiology of kidney stones is considered multifactorial, we conducted the first bidirectional MR analyses to comprehensively estimate the causal effects of 46 potential risk factors on nephrolithiasis risk. The present MR study, in which genetic variants were used as proxies for modifiable risk factors, identified that genetically predicted higher levels of total triglycerides, fasting insulin, serum 25(OH)D, serum calcium, waist-to-hip ratio, and urinary sodium, and lower levels of tea consumption, urine pH, income, education, and serum phosphorus could causally increase the risk of kidney stones (Fig. 3).

Obesity has been extensively investigated to be independently associated with the risk of kidney stone formation [28]. As a general obesity-related index, BMI was proved to causally increase the risk of kidney stones by a previous MR analysis. Still, there was a large sample overlap between the exposure and outcome data (~ 60% from UK Biobank) [10]. Considering that sample overlap might inflate the weak instrument bias and type 1 error rate [29], we excluded individuals from UK Biobank in the BMI data set and found no evidence for the relationship between BMI and kidney stones. In addition, WHR is another important obesity-related indicator, which can reflect the fat distribution and is more likely to unmask the association of obesity with health outcomes [30]. Based on summary statistics from UK Biobank, our MR study provided the first significant evidence for the causality between WHR after adjusting for BMI and nephrolithiasis risk, suggesting central obesity was a more important risk factor in kidney stones than general obesity. The pathophysiologic mechanism responsible for the connection between obesity and stone formation is uncertain. In detail, insulin resistance, commonly associated with obesity, can decrease urine pH and increase stone formation [28]. Other evidence linking obesity and calcium oxalate stones was that obesity-related hyperinsulinemia and insulin resistance could modulate urine composition, such as lower levels of urine pH and citrate and higher levels of oxalate and calcium [28, 31]. Besides, a previous study showed that body fat percentage, as an indicator of visceral fat content, could contribute to the formation of kidney stones in adults aged ≥ 40 yr [30]. Nevertheless, we did not find a causal association between body fat percentage and kidney stones, possibly due to limited LVs and insufficient power in our MR analysis.

In addition to central obesity, metabolic syndrome (MetS) and its other components, including hypertension, hyperglycemia, and dyslipidemia, have been linked to an increased risk of kidney stones [32]. West et al. found that MetS traits were associated with a higher risk of stone history (8.8% vs. 4.3%) compared with health status [33]. Moreover, the risk increased with the number of MetS traits and was a twofold increase in individuals with four or more traits [33]. We found only one published GWAS related to MetS based on the harmonized NCEP criteria [34]. Thus, our MR analysis explored the causal association of MetS with the incidence of kidney stones for the first time and found no causality between them. Due to inconsistent criteria for MetS and the limited power, we think this causality deserves further investigation. Next, we analyzed the association between single metabolic traits and kidney stones. A previous MR study found that T2D was positively associated with kidney stones in the UK Biobank and FinnGen consortium [10]. Given that exposure individuals were from multi-ancestries (~ 21% of non-European) and sample overlap (~ 40% from UK Biobank), we only included statistics from the DIAGRAM consortium without UK Biobank. We revealed suggestive causality between T2D and kidney stones. Interestingly, our MR results showed strong evidence for a positive association of fasting insulin with the incidence of kidney stones in both MR stages. As mentioned above, hyperinsulinemia and its related insulin resistance could decrease urine pH and citrate levels and increase levels of urine oxalate and calcium [28, 31]. Thus, we considered that insulin levels and function might be more important than glycemia in increasing the risk of kidney stones. As the central theme of MetS, the causal relationship between insulin resistance and kidney stones needs to be further directly explored by MR analysis.

Regarding blood lipids, suggestive evidence for a positive association between total triglycerides and kidney stones was found in the FinnGen consortium and UK Biobank, respectively. In contrast, no evidence supported the effect of the total, low-density lipoprotein and high-density lipoprotein cholesterol on kidney stones. Although the potential mechanisms have yet to be elucidated, Torricelli et al. identified that specific dyslipidemia (including high triglycerides) might portend unique alterations in urine compositions and predispose them to kidney stone formation [35]. In a recent meta-analysis, all nine included studies found that a history of kidney stones significantly elevated the risk of hypertension [36]. Reversely, Madore et al. found that hypertension did not affect nephrolithiasis risk in men and wemon [37, 38]. A previous MR analysis reported that kidney stone disease could unidirectionally increase the risk of hypertension with little impact (OR = 1.001) [11]. On the contrary, we verified that the presence of kidney stones was causally connected with high systolic and diastolic blood pressure (OR = 1.51, *P* = 0.0005; OR = 1.28, *P* = 0.0001, respectively), but not vice versa. Combined with our findings, we deemed that regulating blood pressure did not reduce the risk of kidney stones.

The effects of dietary and lifestyle factors on nephrolithiasis have been investigated in several observational studies [39]. In the present MR study, we were the first to systemically explore the relationship between diet- and lifestyle-related risk factors with available GWAS data and nephrolithiasis risk. Caffeinated beverages, including coffee and tea, are the primary sources of dietary caffeine [40]. Many previous studies verified the protective effect of caffeine on kidney stones [40, 41], and a study with 39 participants speculated that caffeine was linked to a higher risk of kidney stones due to its increased urinary calcium excretion [42]. In 2014, the most extensive prospective study, including 217,883 participants from HPFS, NHS I, and NHS II cohorts, showed that participants in the highest quintile of caffeine intake (568 ± 185 mg caffeine/d) had a 26%, 29%, and 31% lower risk of stone development in the above three cohorts, respectively (*P*-trend < 0.001 for all cohorts) [43]. Furthermore, in a larger number of individuals selected from UK Biobank (439,072 participants), the results demonstrated that not only increased intake of tea but also coffee were independently associated with a lower risk of kidney stones [44]. Our MR analysis, which excluded individuals from UK Biobank in exposure data to avoid sample overlap, confirmed the inverse association between consumed caffeine from tea and the risk of kidney stones. Due to inadequate instrumental variables, we could not use the IVW method to investigate the association of coffee consumption with kidney stones. Based on these collective results from the above cohorts and MR analyses, there was convincing evidence that intake of caffeine from tea prevented the incidence of kidney stones. Concerning mechanisms, intake of caffeine could elevate the urinary excretion of citrate to inhibit the formation of calcium oxalate stones and increase the urine volume to reduce the supersaturation of calcium and oxalate ions [40].

In addition, we found that genetically predicted higher income played a protective role in the formation of kidney stones in the FinnGen study, and longer education attainment was proved to reduce nephrolithiasis risk in UK Biobank. However, the possible mediators and mechanisms for these causal associations were not examined by our MR analysis. The reasons might be that the gradients in education or income influenced the existing gradients of dietary behavior, unhealthy lifestyle factors, environmental factors, and cognitive ability to disease, ultimately resulting in disparate stone outcomes [45, 46]. Furthermore, our MR outcomes showed no significant associations of smoking, alcohol consumption, glucocorticoid use, physical activity, sedentary leisure hours, and diet with the risk of kidney stones. However, because of the limited power of these analyses, we cannot preclude these factors from affecting kidney stones, especially dietary factors with no detailed categorization.

Higher calcium supplementation could promote intestinal oxalate availability and urinary excretion and then accelerate the formation of kidney stones [47]. A phenome-wide MR study revealed that serum calcium levels were causally connected with the risk of kidney stones [16]. In addition, one MR from UK Biobank uncovered a positive causality between serum 25(OH)D and kidney stones after adjusting the effect of serum calcium [15]. However, a meta-analysis found that only vitamin D combined with calcium supplementation increased the incidence of kidney stones [48], and a synergistic role of vitamin D and calcium was proved in a rat model of kidney stone disease [49]. Our study optimized the MR design with no sample overlap, two stages, and reverse MR analysis. Our findings suggested that higher serum levels of 25(OH)D and calcium causally led to the elevated risk of kidney stones. Furthermore, higher serum phosphorus levels were proved to predict a protective effect in stone formation in the discovery stage. Calcium and phosphorus homeostasis are well known as essential to human physiology [50]. We speculated that changes in serum phosphorus could directly affect the stone formation or indirectly through serum calcium. Due to lacking individual-level data, we could not perform multivariate MR to adjust the interaction among serum calcium, phosphorus, and vitamin D, which requires further exploration.

A low urine pH, increasing the undissociated form of uric acid, leads to uric acid stone formation and provides nucleation with uric acid crystals to predispose to calcium oxalate stone formation [14]. Our MR study provided the first significant evidence for the negative causality between urine pH and nephrolithiasis risk. Thus, urinary alkalinization and frequent monitoring of urine pH are essential for preventing most stones. Consistent with the outcomes of previous MR [14], which only included UK Biobank data, there was no evidence demonstrating the effect of serum uric acid on the formation of kidney stones in our MR analysis. Furthermore, we conducted reverse MR analysis and still found no causal association. A possible reason was that urate was only involved in the pathogenesis of part types of urolithiasis [14]. Increased sodium intake promotes nephrolithiasis by leading to hypercalciuria and hypocitraturia [51]. At the same time, a retrospective study based on a 24-h urinalysis database revealed that urinary sodium level was negatively associated with urine calcium oxalate supersaturation [52]. On the contrary, the present MR analysis found that a higher urinary sodium level significantly increased kidney stones risk. This discrepancy might be attributable that only stone patients were included in the retrospective study, thus generating a bias when excluding the healthy population.

There are several methodological strengths in our MR study. First, this is the first MR study to investigate modifiable risk factors related to nephrolithiasis comprehensively. Second, even if some included GWASs data overlapped with some in previous MR analyses, and thus our MR outcomes could not be considered independent replication. However, we added additional GWAS data or used the newest summary statistic data of FinnGen consortium and UK Biobank and improved our MR design to interpret the evidence based on the results of three parts, including discovery, validation, and meta-analysis stages, which could add much more confidence to our research. Third, there was no sample overlap between the cases and controls in the discovery and replication stage, thus deflating the weak instrument bias and type 1 error rate [29]. All F-statistics were more than 10 in this MR study. Finally, with available summary statistics, bidirectional MR was conducted on the risk factors. However, several limitations should be noted. First, some analyses' statistical power was limited, as demonstrated in Additional file 1: Table S6–S7. Thus, we cannot exclude type II errors as an explanation for the null associations. In addition, we performed a strict selection procedure of IVs, which could reduce the number of IVs and then decrease the ability to explain the phenotypic variance in the exposure. Therefore, larger GWASs are needed to provide more IVs and adequate power to investigate weak-to-moderate associations. Second, our MR analyses assumed linear associations, whereas nonlinear or J-shaped–curve associations could not be assessed because of lacking individual-level data. Third, the MR approach is less flexible than a cohort study in analyzing the independent associations of multiple exposures. Due to lacking individual-level data, multivariable MR analysis cannot be conducted to adjust for covariates. Forth, our MR analyses were restricted to individuals of European ancestry and thus could not be expanded to other populations. Last, this study could not distinguish the difference among different stone types.

## Conclusions

In conclusion, the present MR study identified higher levels of total triglycerides, fasting insulin, serum 25(OH)D, serum calcium, waist-to-hip ratio, and urinary sodium, and lower levels of tea consumption, urine pH, income, education, and serum phosphorus causally increased the risk of kidney stones. Modulation of these modifiable risk factors can guide the prevention of nephrolithiasis.

## Supplementary Information

**Additional file 1**. Supplementary Tables of causal effects of modifiable risk factors on kidney stones: A bidirectional Mendelian randomization study.

## Data Availability

Only publicly available data were used in this study, and data sources and handling of these data were described in the Materials and Methods and supplementary Table S2–S3. Discovery stage data from the FinnGen consortium can be downloaded from https://doi.org/10.1101/2022.03.03.22271360. Replication stage data from UK Biobank are available at the Pan-UKB team. https://pan.ukbb.broadinstitute.org. 2020. The website (mRnd) for calculating the statistical power: http://cnsgenomics.com/shiny/mRnd/. The PhenoScanner database is used to find IVs associated with other phenotypes (http://www.phenoscanner.medschl.cam.ac.uk/).

## Abbreviations

T2D: Type 2 diabetes
MR: Mendelian randomization
SNPs: Single-nucleotide polymorphisms
GWASs: Genome-wide association studies
IVs: Instrument variables
TSMR: Two-sample MR
MAF: Minor allele frequency
LD: Linkage disequilibrium
ICD: International Classification of Diseases
IVW: Inverse-variance weighted
MR-PRESSO: The MR pleiotropy residual sum and outlier
CI: Confidence intervals
FDR: False discovery rate
SD: Standard deviation
WHR: Waist-to-hip ratio
BMI: Body mass index
MetS: Metabolic syndrome
